# Supplementary material for: Comparative Study of Bacterial Microbiota Differences in the Rumen and Feces of Xinjiang Brown and Holstein Cattle
Source: Animals (Basel). 2024 Jun 10;14(12):1748. doi: 10.3390/ani14121748 (PMC11200985; doi:10.3390/ani14121748)
Supplement: Supplementary file 1 [file animals-14-01748-s001.zip › animals-2998867-supplementary.pdf]

Table S1. Statistics of microbiota between Xinjiang Brown and Holstein cattle at the phylum level.

**Bacterial**	**Relative abundance**	**Relative abundance**
Rumen	Xinjiang Brown	Holstein
Bacteroidetes	0.52	0.54
Firmicutes	0.32	0.28
Proteobacteria	0.12	0.12
Patescibacteria	0.01	0.02
Spirochaetes	0.01	0.02
other	0.02	0.02
Feces	Xinjiang Brown	Holstein
Firmicutes	0.66	0.63
Bacteroidetes	0.29	0.32
Spirochaetes	0.02	0.03
Tenericutes	0.01	0.01
other	0.02	0.01
